# Willingness to pay of Nigerian poultry producers and feed millers for aflatoxin‐safe maize

**DOI:** 10.1002/agr.21621

**Published:** 2019-07-09

**Authors:** Andrew M. Johnson, Tahirou Abdoulaye, Bamikole Ayedun, Joan R. Fulton, Nicole J. Olynk Widmar, Akande Adebowale, Ranajit Bandyopadhyay, Victor Manyong

**Affiliations:** 1 Department of Agricultural Economics, Purdue University, West Lafayette, Indiana; 2 International Institute of Tropical Agriculture, Ibadan, Nigeria; 3 International Institute of Tropical Agriculture, IITA‐Abuja Abuja Station, Kubwa Abuja, Nigeria; 4 International Institute of Tropical Agriculture, Dar es Salaam, Tanzania; 5 Independent Consultant for Deloitte Consulting LLP, United States

## Abstract

Dietary aflatoxin exposure is a widespread problem in the developing world and causes severe negative health consequences to humans and livestock animals. A new biological control product, called Aflasafe, has been introduced in Nigeria to mitigate aflatoxin contamination of maize in the field and in storage. No known prior work has estimated how much African agribusinesses using maize for animal feed will pay for aflatoxin‐safe maize. This study measured the levels of Aflasafe awareness, surveyed current aflatoxin management practices, and estimated, using choice experiments, willingness to pay (WTP) for aflatoxin‐safe maize by Nigerian poultry producers and feed millers. Data was gathered from 272 orally administered surveys, which included discrete choice experiments examining maize purchasing decisions. Results suggest that the proportion of enterprises that were aware of aflatoxin was found to vary across states. Two latent classes of Nigerian poultry producers and feed millers were identified that were willing to pay average premiums of 4.9% and 30.9%, respectively for maize with 10 parts per billion (ppb) aflatoxin concentration relative to maize with 20 ppb aflatoxin concentration. Both latent classes were, on average, willing to pay larger premiums for maize with 4 ppb aflatoxin concentration. There was evidence that latent class membership, and hence WTP, varied based on awareness of aflatoxin and across geographies.

**JEL CLASSIFICATION:**

D29; I15; O13

## 1 INTRODUCTION

Aflatoxin is a potent toxin, predominantly produced by the fungus *Aspergillus flavus*, with severe negative impacts on both human and animal health (Williams et al., 2004). Crops, including maize, can be populated with *A. flavus* and contaminated with aflatoxin both preharvest and postharvest (Bandyopadhyay et al., 2016). Humans are exposed to aflatoxin by consuming contaminated grains, nuts, and legumes or by consuming meat, eggs, or milk from animals with aflatoxin‐contaminated diets (Iqbal, Nisar, Asi, & Jinap, 2014; Keyl & Booth, 1971; Liu & Wu, 2010). Approximately 4.5 billion persons living in developing countries are chronically exposed to largely uncontrolled amounts of the toxin (Williams et al., 2004). Leading consequences of aflatoxin consumption in humans include liver cancer, immunosuppression, stunted child growth, and (in extreme instances) rapid death (Azziz‐Baumgartner et al., 2005; Gong et al., 2002; Turner, Moore, Hall, Prentice, & Wild, 2003; Williams et al., 2004).

Detrimental effects of aflatoxin are observed in livestock fed with rations containing high concentrations of the toxin (Bryden, 2012). Dietary aflatoxin exposure reduced feed conversion efficiency in pigs and poultry by 7–10% (Shane, 1993, as cited in Williams et al., 2004). Aflatoxin has also been linked to immunosuppression in piglets and poultry (Williams et al., 2004) and has been connected to decreased weight gain in pigs (Marin et al., 2002). Aflatoxin consumption can also decrease egg production in layers and increase poultry mortality rates (Shane, 1993). The effects of aflatoxin consumption can be exacerbated if other toxins are also present in feed rations, which is relatively common (Huff, Kubena, Harvey, & Doerr, 1988).

The poultry industry is an important component of Nigerian agriculture. In 2013, Nigerian poultry farmers produced 650,000 metric tons of eggs (the most of any country in Africa) and 169,991 metric tons of chicken meat (fifth most in Africa; FAOSTAT, 2017). The total value of Nigerian poultry industry output in 2013 was estimated at ₦80 billion (Sahel Capital Limited, 2015). Sorting of grain with high aflatoxin concentration away from human consumption and toward animal consumption may contribute to the high rates of contamination of poultry diets (Hoffmann, Mutiga, Harvey, Nelson, & Milgroom, 2013). Ezekiel, Bandyopadhyay, Sulyok, Warth, and Krska (2012) found that 62% of sampled commercially available poultry feeds in Nigeria had aflatoxin concentrations above 20 part per billion (ppb), the maximum allowable concentration level for grains to be considered safe in the United States.

Scientists at the International Institute of Tropical Agriculture (IITA) and the United States Department of Agriculture —Agricultural Research Service together with other national partners have recently developed the biological control product Aflasafe to reduce aflatoxin contamination of maize and groundnut (Bandyopadhyay et al., 2016). The active ingredient of Aflasafe is a mixture of four Nigerian strains of A. flavus incapable of producing aflatoxins (also called atoxigenic strains). When applied in the maize fields before crop flowering, the atoxigenic strains in Aflasafe out‐compete and replace the toxic strains in the field, thereby reducing aflatoxins in the crop. If adopted widely by farmers, Aflasafe usage could result in significantly higher volumes of verified aflatoxin‐safe maize (i.e., maize that is safe for human and animal consumption) being available in Nigerian markets than is available currently (Bandyopadhyay et al., 2016). Greater availability of aflatoxin‐safe maize could produce substantial direct benefits to Nigerian poultry farmers by mitigating the damages of aflatoxin‐contaminated feed described above. Furthermore, there could be substantial spillover benefits to the farmers and feed millers providing maize and feed inputs to poultry farmers and to the consumers of the eggs and meat produced by Nigerian poultry farmers.

Research has been done to evaluate consumer WTP for aflatoxin‐safe maze. Recent experimental work shows that Kenyan consumers are willing to pay premiums for verified aflatoxin‐safe maize (De Groote et al., 2016;

Hoffmann & Gatobu, 2014). Despite a positive mean WTP for aflatoxin‐safe maize, WTP differed between geographical regions and aflatoxin knowledge. De Groote et al. (2016) found that consumers in the driest geographic regions of their sample in Kenya, where aflatoxicosis outbreaks were most common, were willing to pay the largest premium for tested, aflatoxin‐safe maize relative to clean maize from the market. De Groote et al. (2016) also found that Kenyan consumers who knew that aflatoxin was toxic were not statistically willing to pay more for tested, aflatoxin‐safe maize than consumers without prior knowledge. However, consumers who knew that aflatoxin was toxic had lower WTPs for untested market maize than consumers who did not previously have that knowledge. De Groote et al. (2016) provided an information treatment to half of the consumers, explaining that consuming large quantities of aflatoxin can cause death and that chronic aflatoxin exposure can lead to liver cancer. De Groote et al. (2016) found the effects of providing an aflatoxin information treatment to be similar to the effects of knowledge of whether aflatoxin was toxic. Consumers that received the treatment were not willing to pay more for tested maize than consumers who did not receive the treatment. But consumers that received the information treatment had lower WTPs for untested maize than consumers who did not receive the treatment. The combined Nigerian agribusiness and Kenyan consumer results may suggest that when individuals become knowledgeable of aflatoxin, they respond by discounting unverified maize in addition to or rather than by offering premiums for verified aflatoxin‐safe maize.

Building on the consumer work, this analysis sought to estimate how much of a price premium Nigerian poultry producers and feed millers (collectively referred to in this article as “agribusiness enterprises”) will pay for aflatoxin‐safe maize. Specifically, the objectives of this paper were (a) to quantify the levels of awareness about aflatoxin and Aflasafe and the levels of understanding of aflatoxin management among poultry producers and feed millers in Nigeria in the fall of 2016; and (b) to estimate the willingness to pay (WTP) of Nigerian agribusiness enterprises for aflatoxin‐safe maize.

## 2 ADDRESSING THE AFLATOXIN PROBLEM

Most countries set food and feed limits for the combined levels of the four forms of aflatoxin: B_1_, B_2_, G_1_, and G_2_ (Food & Agriculture Organization [FAO], 2004). Regulations for permissible levels of aflatoxin in human food vary across countries. The European Union’s (EU’s) limit for cereals, groundnuts, oilseeds, almonds, and pistachios used for human consumption is 4 ppb (European Commission, 2016). The United States (US) has a less‐stringent standard for total aflatoxin concentration of 20 ppb for any raw food products for human consumption (Mitchell, Bowers, Hurburgh, & Wu, 2016). Other countries establish standards that fall between the EU and the US. For example, Ghana and Kenya have limits of 15 and 10 ppb, respectively (Gajate‐Garrido, Hoffmann, Magnan, & Opoku, 2016). Nigeria’s standard is 10 ppb (Adetuniji et al., 2014).

When regulations are well enforced, as in most developed countries, aflatoxin problems are generally well controlled (Bandyopadhyay, Kumar, & Leslie, 2007). However, the cost of complying with regulation is substantial (Xiong & Begin, 2012). Furthermore, enforcement is often weak or difficult in developing countries. De Groote et al. (2016) conducted a welfare analysis on the costs and benefits of mandatory testing of all maize in Kenya. They found that aflatoxin testing increased economic surplus if the cost of testing was reasonable, if administrative costs were minimal, and if only small amounts of maize were discarded for testing high in aflatoxin concentrations.

The AgResults Nigeria Aflasafe pilot project was launched in 2013 to help overcome barriers to widespread market adoption of Aflasafe (AgResults Initiative, 2017). The pilot project works with private farm‐based businesses, called implementers that purchase and distribute Aflasafe to their constituent farmers and aggregate the resulting production of aflatoxin‐safe maize (AgResults Initiative, 2017). The pilot project makes an incentive payment of US $18.75 for every metric ton of high‐Aflasafe maize (i.e., at least 70% of *Aspergillus* strains in the grain should belong to one of the four constituent strains of Aflasafe) that the implementers aggregate for sale (AgResults Initiative, 2017). In the typical range of market prices of US $250—375 per ton of maize and average yield of 2.6 tons maize grains per ha, this incentive payment constitutes an effective premium of 5–13%, the long‐term project benefit of aflatoxin‐safe maize over the maize currently offered in the market (AgResults Initiative, 2017). The pilot project does not take possession of the maize; it only makes the US $18.75 incentive payment for the maize tonnage categorized as high‐Aflasafe maize. The implementers retain possession of the maize for sale in the open market. As of December 2016, the implementers with verified low‐aflatoxin maize were receiving a premium even higher than US $18.75 per metric ton in private transactions in the market (Bandyopadhyay et al., 2016).

The premium in private transactions may be from buyers channeling maize toward either human consumption or animal feed. Recent analyses in Kenya have estimated that consumers will pay premiums of 7.4–24% for verified aflatoxin‐safe maize compared with maize that is clean but had not been tested for aflatoxin (De Groote et al., 2016; Hoffmann & Gatobu, 2014). This paper on Nigerian agribusinesses builds on the literature by examining the WTP of maize buyers who are expected to channel the maize toward farm animal, rather than human, consumption.

## 3 MATERIALS AND METHODS

### 3.1 Data collection and survey instrument

**Figure 1:**
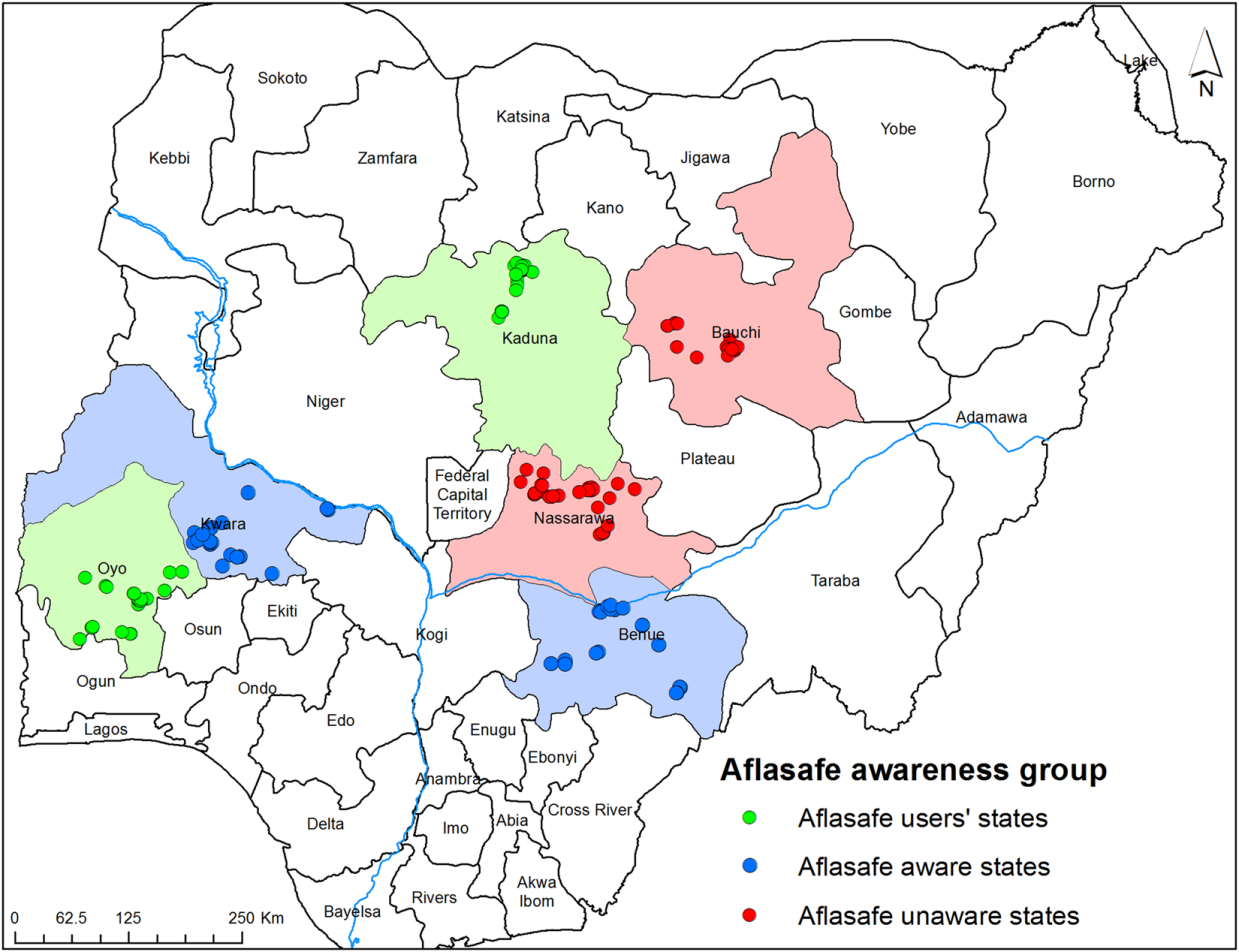
Map of Nigeria with locations of Agribusinesses having the various status of Aflasafe awareness [Color figure can be viewed at wileyonlinelibrary.com]

Researchers developed a survey, which incorporated choice experiment methods, to collect primary data for this analysis. Survey enumerators were trained during September 28–29, 2016, in Abuja, Nigeria. All 15 enumerators held a bachelor’s degree, and some had more advanced degrees. The surveys were conducted during October and November of 2016 in six Nigerian states, with precise survey locations identified in Figure 1.

There were three classes of agribusiness enterprises: (a) those from Benue and Kwara States where there is awareness of Aflasafe but Aflasafe is not used; (b) those from Kaduna and Oyo States where there is awareness of Aflasafe and Aflasafe is used; and (c) those from Nasarawa and Bauchi States where there is generally low awareness of Aflasafe and Aflasafe is not used. The Farmer Association of Nigeria provided a list of poultry farmers and feed millers in each State except Nasarawa, where the list was provided by the Nasarawa State Agricultural Development Program (ADP). Survey enumerators targeted 50 randomly selected (from the lists provided) poultry farmers and feed millers in each state. In Bauchi and Nasarawa States, many poultry farms were no longer operating due to a shortage of maize grains and other factors. Consequently, 50 poultry farmers and feed millers could not be obtained from each of the states. The following numbers were obtained from each state: 51 in Oyo State, 47 in Kaduna State, 50 in Kwara State, 51 in Benue State, 28 in Bauchi State, and 45 in Nasarawa State. The number of valid poultry farmers from all states was 272.

The survey received an Internal Review Board approval from the affiliated institutions. Enumerators explained (verbally) to respondents that participation was voluntary and received verbal consent before proceeding with surveys. Responses to questions were recorded in CSPro 6.3. After collecting responses to the discrete choice experiment, respondents were asked 21 questions about the characteristics of their enterprises. The agribusiness survey, including the choice experiment, is provided as part of the supplementary information in an online appendix.

### 3.2 Choice experiment

Each agribusiness participant was presented with one of two blocks of seven trinary choice sets. Two of the alternatives in each choice set involved the agribusiness purchasing maize with a verified level of aflatoxin; the third alternative was always an “Optout,” where the agribusiness could choose to not purchase either aflatoxintested maize option provided (and instead purchase regular maize with an unknown concentration of aflatoxin contamination at the current market price). Non‐opt out alternatives presented information about two attributes of interest in this analysis, including the level of aflatoxin concentration in the maize and the percentage premium the agribusiness must pay over the regular market price. While two is a smaller number of attributes compared with many choice experiments, the small number offers the advantage of reduced decision complexity. Parameter estimates in choice experiments have been shown to be sensitive to the complexity of choice tasks (DeShazo & Fermo, 2002; Swait & Adamowicz, 2001).

There were three possible levels of aflatoxin concentration in maize (20, 10, and 4 ppb), which corresponded to the maximum US, Nigerian, and European thresholds for using maize to produce human food. There were six possible price premiums for the maize within the choice experiment, namely 0%, 1–10%, 11–20%, 21–30%, 31–40%, and 41–50%. Choice sets were generated by the OPTEX procedure in SAS to be orthogonal and optimize D‐efficiency.

### 3.3 Methodology

Econometric estimation used in this analysis is based on random utility theory. According to Lancaster (1966), consumers derive utility not directly from the goods they consume but rather from the specific attributes of the goods they consume. Hence, consumer *i*’s utility (*U*_*ij*_) from consuming product *j* is defined in Equation (1). Product *j* must be selected from a finite choice set. *V*_*ij*_ is the contribution to utility of all observed factors, including the pertinent attributes of product j (Hensher, Rose, & Greene, 2005). *ε*_*ij*_ is the contribution to utility of all unobserved factors and is assumed to be independent of and additive to *V*_*ij*_ (Hensher et al., 2005).

$$U_{ij}=V_{ij}+\varepsilon_{ij}$$ 1

Consumers are assumed to maximize utility. Thus, when consumer *i* is presented with *j* = 1, …, *J* alternatives, he/she is assumed to choose the alternative with the greatest utility (*U*_*ij*_). Because the *ε*_*ij*_ portion of Uij is unobserved, it is not possible to deterministically identify the greatest *U*_*ij*_ before a choice is made. However, it is possible to estimate the probability with which a consumer will choose each given alternative from only the observed information (*V*_*ij*_). Hensher et al. (2005, pp. 82) explain, “the probability of an individual choosing alternative *l* is equal to the probability that the utility of alternative *l* is greater than (or equal to) the utility associated with alternative *j* after evaluating each and every alternative in the choice set of *j* = 1, … *l*, … J alternatives.” This statement is expressed as follows, where Prob_*il*_ is the probability that consumer i chooses alternative *l*.

$${\text{Prob}}_{il}=\text{Prob}[(V_{il}+\varepsilon_{il})>(V_{ij}+\varepsilon_{ij})\;\forall \;j\in j=1,\ldots ,J;j\neq l].$$ 2

Train (2009) shows that if ε_ij_ from Equation (1) is treated as random and assumed to be independently and identically distributed extreme value, then Probil can be rewritten in Equation (3).

$${\text{Prob}}_{il}=e^{vil}/\sum_{j=1}^{J}e^{V_{ij}.}$$ 3

Equation (3) is the specification for the multinomial logit model (MNL), which allows for an estimation of consumer *i*’s decision based only on the observed information.

Utility is most often assumed to be linear in parameters (Jones, Alexander, Widmar, Ricker‐Gilbert, & Lowenberg‐DeBoer, 2016. This allows *V*_*ij*_ to be further specified in Equation (4). Where **X***ij* is a vector of observable information of product attributes and **β** is a vector of parameters to be estimated.

$$V_{ij}{\text{ = }\beta^{\prime }\text{X}}_{ij}=\;\beta_{1}(\text{Premium})+\beta_{2}(\text{OptOut})+\beta_{3}(10\text{ ppb})+\beta_{4}(4\text{ ppb}).$$ 4

“Premium” is the percentage difference between the maize pricefor agiven alternativeand the price of regular market maize. It was measured at the midpoints of each range presented at the end of Section 3.2. “OptOut” is a binary variable indicating whether the third option in a choice set was selected. “10 ppb” is an effects‐coded trinary variable taking value 1 for maize verified with 10 ppb aflatoxin, value −1 for maize with 4 or 20 ppb aflatoxin, and value 0 for the OptOut. “4 ppb” is effects‐coded in a similar manner, taking value 1 for maize verified with 4 ppb aflatoxin. The effects‐coded variable “20 ppb” was omitted from the model to avoid the dummy variable trap. Effects coding prevents a variable’s effects from being confounded with the “OptOut” effects (Tonsor, Olynk, & Wolf, 2009).

Utility maximization within consumer theory is presented here because it is the foundation on which WTP literature is typically built (Olynk, Wolf, & Tonsor, 2012). Notwithstanding, Lusk and Hudson (2004) showed that the concept can easily be extended to profit maximization within the theory of the firm by demonstration that a producer’s WTP for a new technology is equal to the difference in the producer’s profit before and after adopting the technology. Multiple authors have applied this framework and methodology to US agricultural production practices of crops (Norwood, Luter, & Massey, 2005), swine (Davis & Gillespie, 2007; Roe, Sporleder, & Belleville, 2004), beef (Norwood, Winn, Chung, & Ward, 2006), and dairy (Olynk et al., 2012; Schulz & Tonsor, 2010).

The most critical shortcoming of the MNL model is that it treats individuals’ preferences as being homogeneous across respondents (note the lack of an i subscript on the **β** in Equation (4); Hensher et al., 2005). One approach to account for heterogeneity is latent class (LC) modeling, which segments a sample into “classes,” and generates separate parameter estimates for each class. LC models allow for heterogeneity between classes but assume homogeneity within each class. A key advantage of this approach is that it can reveal the size and tastes of different segments within a market (Louviere, Hensher, & Swait, 2000).

Equations (5) and (6) extend Equation (3) to show how to estimate the probability that individual *i* selects alternative *l* under an LC model (Louviere et al., 2000). Individuals are divided into S classes and the probability of individual *i* falling into class s is given by *W*_*is*_. It is impossible to simultaneously estimate the *λ*_*s*_ scale factors and *β*_*s*_ coefficients for each class (Louviere et al., 2000). This analysis makes the typical assumption that every λs is equal to one (Lusk, Roosen, & Fox, 2003).

Probil/s=eλsβ′sXil∑j=1Jeλsβ′sXij, 5

$${\text{Prob}}_{il}=\sum_{S=1}^{S}{\text{Prob}}_{il/s}W_{is}.$$ 6

### 3.4 Estimated agribusiness enterprise WTP

Given the assumption of linearity, the *β* parameters are the marginal utilities of the product attributes in **X**_*ij*_. Incremental changes in utility do not map to incremental changes in behavior, because utility is an ordinal measure. However, if one of the product attributes in **X**_*ij*_ is price, then price’s marginal utility can serve as a basis for estimating the monetary value of the other variables, referred to as WTP. Equation (7) shows the standard way of estimating WTP for a given product attribute, *k* (Hensher et al., 2005).

$${\text{WTP}}_{k}=-\frac{\beta_{k}}{\beta_{1}},k\in 2,3,4.$$ 7

For this analysis, the subscripts on the *β* coefficients in Equation (7) correspond to the subscripts in Equation (4). Effects coding was used for the 10 ppb and 4 ppb variables in Equation (4), necessitating the WTP estimates for these two variables from Equation (7) to be scaled‐up by a factor of 2 (Lusk et al., 2003).

As described by Lusk and Hudson (2004), producers are indifferent between using two input bundles that provide the same level of profit. The ratio of marginal utilities in Equation (7) is the number of currency units the producer can give up in exchange for one unit of k and maintain the same level of profit. Hence, the ratio measures the amount of currency at which the producer is indifferent to between the currency and one unit of *k*. This analysis estimates mean WTP values and confidence intervals using the Krinsky‐Robb method (1986).

## 4 RESULTS

### 4.1 General sample group characteristics

Summary statistics for the sample group are presented in Table 1. The sample was not representative of the full national population of Nigerian “agribusiness enterprises” (i.e., poultry farmers and feed millers). Enterprises were classified in mutually exclusive categories based on the types of products produced: poultry only, feeds only, or poultry and feeds. Furthermore, enterprises were divided into three mutually exclusive scale classes based on the annual volume of maize used. Small‐scale operations used less than 10 tons of maize per year and constituted 60% of the agribusinesses in the sample. Medium scale operations used between 10 and 100 tons per year and represented 30% of the agribusinesses. Large‐scale operations used over 100 tons of maize per year and made up 10% of the agribusinesses. In addition to questions about production and scale, respondents were also asked a series of questions about the demographic characteristics of the head of the enterprise and about the attributes of the business.

**TABLE 1:** Summary Statistics of Sample Group, *n* = 272

	Mean	Median	SD	Min	Max
Enterprise years in operation	8.9	7	7.7	1	50
Age of head of enterprise	45.1	44	11.7	23	90
Years of education of enterprise head	15.8	16	2.5	0	21
					Count
Enterprises with a female head					44 (16%)
Enterprises registered with local or state government					166 (61%)
Enterprises registered with NAFDAC					22 (8%)
Enterprises belonging to a professional poultry association					145 (53%)
Enterprises that are sole proprietorships					239 (88%)
Enterprises with access to microcredit					69 (25%)
Enterprises that were implementer with AgResults					21 (8%)
Enterprises that work with an AgResults implementer					4 (1%)
Enterprises producing only poultry					147 (54%)
Enterprises producing poultry and feeds					108 (40%)
Enterprises producing only feeds					17 (6%)
Small scale enterprises (<10 tons maize/year)					162 (60%)
Medium scale enterprises(10–100 tons maize/year)					83 (31%)
Large scale enterprises (>100 tons maize/year)					27 (10%)

Note: Count as percentage of full sample group in parentheses.

Abbreviation: NAFDAC, National Agency for Food and Drug Administration and Control.

The mean number of years in business for the sample of agribusinesses studied was approximately 9 years, while the median of the sample was 7 years. Enterprises were registered with local or state governments at a much higher rate (61%) than with NAFDAC (8%). Approximately one‐quarter of enterprises had access to microcredit and 82% of the enterprises were organized as sole proprietorships.

Twenty‐one enterprises (7.7% of sample) were an implementer with the AgResults Nigeria Aflasafe pilot project at the time that they participated in the survey. Sixteen of these 21 operations were in Oyo, Kwara, or Kaduna States where the pilot project was most active as of October 2015 (AgResults Initiative, 2015). Four enterprises reported doing business with an implementer with the pilot project, suggesting that they use the implementer as a supplier of maize grain.

### 4.2 Aflatoxin and Aflasafe awareness and management

The awareness of aflatoxin and Aflasafe among the respondents and about their enterprise’s current aflatoxin management strategies are reported in Table 2. There is clear variation in the level of aflatoxin awareness among states in this sample. The southwestern states of Oyo and Kwara had awareness levels statistically and substantially higher than any of the other states.

**TABLE 2:** Detailed decomposition of the percent of agribusinesses with or without awareness of aflatoxin and Aflasafe, and using or not using aflatoxin and management practices

	Have you heard of aflatoxin?		Do you control for aflatoxin?		Have you heard of Aflasafe?	
	Yes (%)	No (%)	Yes (%)	No (%)	Yes (%)	No (%)
Full sample. *n*=272	42.3	57.7	31.6	68.4	12.9	87.1
*Decomposed by state*						
Oyo. *n*=51	92.2^a^	7.8	92.2^a^	7.8	37.3^a^	62.7
Kwara. *n*-50	70.0^b^	30.0	46.0^b^	54.0	10.0^b^	90.0
Bauchi. *n*-28	32.1^c^	67.9	17.9^c^	82.1	0.0^bc^	100.0
Benue. *n*=51	23.5 ^cd^	76.5	17.6^c^	82.4	11.8^b^	88.2
Nasarawa, *n*=45	15.6 ^cd^	84.4	2.2^d^	97.8	0.0^c^	100.0
Kaduna, *n*=47	10.6^d^	89.4	2.1^d^	97.9	10.6^b^	89.4
*Decomposed by type of products made*						
Poultry alone, *n*=147	34.7^a^	65.3	25.1^a^	74.9	12.9^a^	87.1
Poultry and feeds. *n*=108	47.2^b^	52.8	32.4^ab^	67.6	12.0^a^	88.0
Feeds alone, *n*-17	76.5^c^	23.5	52.9^b^	47.1	17.6^a^	82.4
*Decomposed by scale of enterprise*						
Small scale	37.7^ab^	62.3	25.3^ab^	74.7	11.1^a^	88.9
(<10 Tons). *n*=162						
Medium scale	51.8^c^	48.2	36.71	63.3	12.0^ab^	88.0
(10-100 tons). *n*=83						
Large scale	40.7^bc^	59.3	321^bc^	67.9	25.9^c^	74.1
(>100 tons). *n*-27						
*Decomposed by registration with iocai or state government*						
Is registered. *n*=166	50.6^a^	49.4	38.0^a^	62.0	16.3^a^	83.7
Is not registered. *n*=106	29.2^b^	70.8	21.7^b^	78.3	7.5^b^	92.5
*Decomposed by membership in professional poultry association*						
Is a member. *n*=145	53.1^a^	46.9	42.8^a^	57.2	18.6^a^	81.4
Is not a member. *n*=127	29.9^b^	70.1	18.9^b^	81.1	6.3^b^	93.7
*Decomposed by awareness of anatoxin*						
Has heard of aflatoxin. *n*=115			74.8%^a^	25.2	26.1^a^	73.9
Has not heard of aflatoxin. *n*=157			0.0%^b^	100.0	3.2^b^	96.8

*Note*: Within each decomposition within each column, numerals with a different letter in superscript (a, b, c, and d) are statistically different from each other (*p* < .05).

Forty‐two percent of respondents had heard of aflatoxin, which is less than the percentage of poultry farmers who were reportedly aware of aflatoxin in 2005 in Benin (65.9%) and Ghana (81.6%; James et al., 2007). In an analogous survey administered to Nigerian maize farmers concurrently with this survey, 72% of respondents had heard of aflatoxin (Johnson et al., 2018). Fewer agribusiness respondents had heard of Aflasafe (12.9%) compared with 67% of maize farmers in a farmer sample that had heard of Aflasafe on the analogous survey (Johnson et al., 2018).

Enterprises that produced feed and poultry had a higher level of aflatoxin awareness than enterprises that produced only poultry. Enterprises that produced only feed had a higher level of aflatoxin awareness than enterprises that produce both feed and poultry. Enterprises that were registered either with local or state government had higher levels of aflatoxin awareness in this sample than enterprises not registered. Similarly, enterprises that were members of poultry associations had higher levels of awareness than enterprises that were not members.

The relative patterns of aflatoxin awareness generally corresponded to whether enterprises control for aflatoxin or not and to whether enterprises had heard of Aflasafe or not. For example, enterprises in states with the highest levels of aflatoxin awareness also controlled aflatoxin in their feed supply at higher rates than other states. It is worth noting that almost three‐quarters of enterprises that were aware of aflatoxin also controlled for it.

Only 10 enterprises (3.7% of sample) tested the level of aflatoxin in their maize supply at the time of the survey. However, 86 enterprises (31.6% per Table 2) made an effort to control aflatoxin contamination. The most frequently cited method of “controlling for aflatoxin” was adding a toxin binder to the feed ration, which was used by 65 enterprises (24.9% of the sample). Certain clay minerals will chemically bind to aflatoxin and reduce the amount of aflatoxin absorption by the gastrointestinal system (Wielogórska, MacDonald, & Elliot, 2016). Sixty‐one of the 65 enterprises used toxin binder alone. Four of the 65 enterprises used toxic binder and an additional control strategy, such as other feed additives or drying maize.

### 4.3 WTP

While efforts were made to analyze various enterprise types, the relatively small number of feed only enterprises makes modeling WTP by enterprise type infeasible. Thus, analysis of the choice experiment was conducted for the data set as a whole. Results of the multinomial logit and LC models are presented in Table 3 and were conducted in NLOGIT 5.0. Multinomial parameter estimates are based on Equations (3) and LC parameter estimates are based on Equation (5). Coefficient estimates have little direct interpretive value; however, the ratios of the coefficient are useful for estimating WTP (Olynk, Tonsor, & Wolf, 2010). The variables “Premium,“ “OptOut,” “10 ppb,” and “4 ppb” estimates are the *β* coefficients from Equation (4). Recalling that the case of maize with an aflatoxin concentration of 20 ppb is omitted from the model, the estimates for “10 ppb” and “4ppb” are relative to maize with 20 ppb aflatoxin concentration.

The sample is divided into two latent classes, with associated class probabilities of 81.8% and 18.2% (Table 3). The two‐class model was selected over models with more latent classes through combined analysis of Akaike information criteria and class size. The makeup of latent classes in terms of individuals is unknown and membership in any once class can only be interpreted probabilistically. Variables can be included in the LC model to help characterize the latent classes by providing information regarding whether that variable increases or decreases the probability of class membership (Widmar, Byrd, Wolf, & Acharya, 2016). The AtoxHear and Southwest dummy variables were statistically significant covariates for characterizing class membership. Covariates that were tested to characterize class make‐up but not found significant included: (a) whether an enterprise was a member of the AgResults Nigeria Aflasafe pilot project, (b) scale of enterprise, (c) type of product produced, (d) ownership structure, (e) registration status with local or state government, (f) membership in professional poultry associations, (g) years of education of respondent, and (h) number of years the enterprise had been operating.

**TABLE 3:** Multinomial logit and latent class coefficient estimation for agribusiness WTP for verified, aflatoxin‐reduced maize

Variable	Description	Multinomial logit	Latent Class 1	Latent Class 2
Premium	% increase over untested market maize	−6.16***	−5.58***	−14.56***
		(0.32)	(0.41)	(1.54)
OptOut	Rejecting both alternatives in choice set	−3.51***	−5.70***	−1.82***
		(0.12)	(0.29)	(0.22)
10 ppb	Maize with 10 ppb aflatoxin concentration	0.71***	0.86***	0.35***
		(0.05)	(0.07)	(0.13)
4 ppb	Maize with 4 ppb aflatoxin concentration	1.39***	1.65***	0.78***
		(0.06)	(0.08)	(0.16)
Constant			2.73***	Fixed
			(0.38)	
AtoxHear	1 = Has heard of aflatoxin		−1.03**	Fixed
			(0.46)	
Southwest	1 = Located in Oyo or Kwara States		−1.29***	Fixed
			(0.46)	
*N*=272	Class probability		81.6%	18.4%
	Log likelihood	−1,216.4	−986.7	
	Akaike information criterion	2,440.9	1,995.4	

*Note*: Standard errors are in parentheses; *** and ** denote significance at the 1% and 5% levels, respectively.

WTP estimates were calculated using parameter estimates in Table 4. WTP confidence intervals were determined using 1,000 bootstrap draws as defined by Krinsky and Robb (1986). Enterprises in both latent classes were willing to pay a bigger premium for maize with 10 ppb aflatoxin concentrations compared with maize with 20 ppb concentrations. This conclusion is based on that fact that in both latent classes the 95% confidence intervals for “10 ppb” do not cross zero. Furthermore, enterprises in both latent classes were willing to pay a higher premium for maize with 4 ppb aflatoxin concentration than maize with 10 ppb concentration. For Class 1, the 95% confidence intervals for “4 ppb” and “10 ppb” do not overlap. While the confidence intervals do overlap for Class 2, the conclusion of statistical differences in means at the 5% level is noted in footnote (a) of Table 4.^1^ The negative WTP’s for opting‐out in both latent classes indicate that enterprises have a preference for having aflatoxin‐reduced maize in their choice set.

**TABLE 4:** Mean willingness to pay (WTP) for Aflatoxin‐verified Maize for each Latent Class

Variable	Description	Latent Class 1	Latent Class 2
OptOut	Rejecting both alternatives in choice set	−102.6%**	−12.6%**
		[−117.2%, −89.4%]	[−15.7 %, −9.9%]
10 ppb	Maize with 10 ppb aflatoxin concentration	30.9%**	4.9%**
		[25.7%, 37.0%]	[1.0%, 8.3%]^a^
4 ppb	Maize with 4 ppb aflatoxin concentration	59.2%**	10.8%**
		[51.5%, 68.0%]	[7.2%, 14.7%]^a^
Class probability		81.6%	18.4%

Note: Units are percentage premiums, interpreted relative to the omitted case of maize with 20 ppb aflatoxin concentration. Values in parentheses signify [lower 95% confidence bound of WTP, upper 95% confidence bound of WTP].

Means and confidence intervals identified using 1,000 Krinsky‐Robb (1986) bootstrap draws.

** Significance at the 5% level.

a Even though the “10 and 4 ppb” confidence intervals overlap for Class 2, the difference between “4 and 10 ppb” in Class 2 was statistically >0 at the 95% confidence level.

The confidence interval for 10 ppb in Class 1 does not overlap the confidence interval for 10 ppb in Class 2. Likewise, the confidence intervals for 4 ppb do not overlap. Therefore, enterprises in Class 1 were willing to pay higher premiums than enterprises in Class 2. Furthermore, enterprises in Class 1 were harmed more by deferring to regular market maize than enterprises in Class 2, as shown by the lower and negative mean “OptOut” estimate for Class 1.

The specific make up of individual classes within any latent class model cannot be determined due to the nature of the model/classes. However the probability that each respondent belongs to a particular latent class can be estimated at the individual respondent level with the specifications of the model. For each individual, the sum of the probabilities that he or she is in each latent class is one. For Table 5, individual respondents were placed into the latent class to which they had the highest estimated probability of belonging. Although not an exact measure of class membership, which is not possible, using the probability of membership is useful for more clearly understanding how the market of agribusinesses purchasing aflatoxin‐reduced maize is segmented. By this method, 81.25% (221 of 272) of respondents were placed in Class 1 and 18.75% (51 of 272) were placed in Class 2. While not exact, these proportions are approximately equal to the more accurate class probabilities, 81.8% for Class 1 and 18.2% for Class 2, in Tables 3 and 4.

**TABLE 5:** Estimated agribusiness latent class demographic characteristics

Count of Respondents	Class 1	Class 2	Full Sample
State			
Oyo	38	13	51
Kwara	26	*24*	50
Bauchi	26	*2*	28
Benue	45	6	51
Nassarawa	44	1	45
Kaduna	42	5	47
Total	221	51	272
Heard of Aflatoxin?			
Yes	76	39	115
No	145	12	157
Total	221	51	272
Control for Aflatoxin?			
Yes	56	30	86
No	165	21	186
Total	221	51	272
**Proportion of respondents in each class**	**Class 1**	**Class 2**	**Total**
By geography			
Oyo or Kwara state, *n* = 101	63%	37%^a^	100%
Other four states, *n* = 171	92%	8%^b^	100%
By awareness			
Had heard of aflatoxin, *n*= 115	66%	34%^a^	100%
Had not heard of aflatoxin, *n* = 157	92%	8%^b^	100%
By control			
Did control for aflatoxin, *n* = 86	65%	35%^a^	100%
Did not control for aflatoxin, *n* = 186	89%	11%^b^	100%

*Note*: Within each decomposition, a and b are statistically different (p < .01); Latent class membership is estimated probabilistically, not deterministically. For the purposes of this table, individuals were assigned to the latent class to which they had the highest probability of belonging; Geography and aflatoxin awareness were found to be statistically significant covariates for explaining latent class membership. Controlling for aflatoxin was not found to be a statistically significant covariate.

As shown by the proportions of enterprises in each class in Table 5, a higher proportion of respondents estimated to belong to Class 2 came from the southwestern states of Oyo and Kwara than of respondents in Class 1. It should be noted, however, of respondents from Oyo and Kwara, a greater proportion was still estimated to belong to Class 1 than to Class 2. A higher proportion of enterprises whose representatives had heard of aflatoxin were estimated to belong to Class 2 than of enterprises whose representatives had not heard of aflatoxin. Similarly, a greater proportion of enterprises controlling for aflatoxin in their feed supply was estimated to belong to Class 2 than of enterprises not controlling.

## 5 DISCUSSION

Less than half of agribusiness enterprise representatives (42.4%) had heard of aflatoxin, and only 13% of enterprise representatives had heard of Aflasafe. Awareness is the first step in the consumer adoption process (Littler, 2015). A decision maker needs to know about a product before he or she can do anything with it. More information about aflatoxin and Aflasafe needs to be disseminated to the maize processing stage of the value chain.

Enterprises in some states were relatively more aware of aflatoxin than in other states. At the high end, 92.2% of respondents in Oyo State had heard of aflatoxin, while only 10.6% of respondents in Kaduna State had heard of aflatoxin. It is noteworthy that Kaduna State had the lowest level of aflatoxin awareness even though implementers in Kaduna State were actively enrolled in the AgResults Nigeria Aflasafe pilot project in October of 2015 (AgResults Initiative, 2015). Agribusiness awareness levels across states do not seem to be following the rollout of the pilot project. AgResults Nigeria Aflasafe pilot project creates market linkages through innovation platforms for both the agricultural enterprises that produce the aflatoxin‐reduced maize under the project and the feed and food industries that are interested in the quality maize. Most of the food and feed industries that use the maize are concentrated in the southern part of Nigeria. Few feed and food industries from the northern part of Nigeria participated in the Innovation Platform.

Differences across states in regard to sophistication in controlling for aflatoxin seemed to parallel differences across states in having heard of aflatoxin. For example, in the southwestern states of Oyo and Kwara, 92.2% and 46.0% of enterprises, respectively controlled for aflatoxin in maize supplies at the time of the survey, the highest rates of any states. These are the same two states with the highest percentages of respondents who had heard of aflatoxin. In Nasarawa and Kaduna States (states with low relative rates of having heard of aflatoxin), just over 2% of the enterprises controlled for aflatoxin. Overall, just under one‐third of enterprises controlled for aflatoxin in maize supplies, typically using toxin binder. A smaller percentage of poultry only enterprises heard of and controlled for aflatoxin than feed only enterprises, although it should be noted that only 17 respondents indicated that they produced only feed.

Given differences observed in aflatoxin awareness between states and the types of products enterprises were making, there may be opportunities to target education to specific geographic regions. Furthermore, efforts to promote awareness of aflatoxin may benefit from targeting managers of poultry only enterprises, since they heard of aflatoxin at a lower rate than feed only enterprises and enterprises producing poultry and feed in the sample collected.

The levels of aflatoxin and Aflasafe awareness were higher among enterprises registered with local or state governments than enterprises not registered. Furthermore, enterprises that were members of a professional poultry association had higher awareness levels than enterprises that were not members. While not conclusive, this data could suggest that these associations are helping to disseminate information about aflatoxin. Obidike (2011) describes state and local government agencies in Nigeria, like ADP, as important providers of information to farmers. Thuo et al. (2014) showed that social network factors, especially weak ties with external support groups such as researchers and extension agents, positively influenced the spread of information about new technology among Ugandan and Kenyan groundnut farmers.

Only 4% of enterprises tested the level of aflatoxin in their maize supply. A cultural change will be needed to get to the point where agribusiness enterprises are controlling for aflatoxin (which is needed for improved health). Testing will be one of the first steps. Testing must be simple, economical, and accessible as a first step for reaping the benefits from aflatoxin control (De Groote et al., 2016).

The WTP results provide evidence that Nigerian feed millers and poultry farmers benefit from purchasing aflatoxin‐reduced maize, even at a price premium. Enterprises in both latent classes were willing to pay premiums for verified aflatoxin‐reduced maize. Strong agribusiness WTP could have beneficial spillover effects on other parts of the Nigerian maize value chain, especially farmers that may be able to receive higher prices for their maize production. As identified by De Groote et al. (2016) cost‐effective testing of the aflatoxin levels in maize will be needed for a market for aflatoxin‐safe maize to function efficiently.

Geography was found to statistically contribute to latent class WTP membership. Respondents in the southwestern states of Oyo and Kwara (where agribusiness awareness levels of aflatoxin were highest) had a higher probability of being in Class 2 with the lower mean WTPs than respondents from other states. This result was robust to including aflatoxin awareness as another covariate for characterizing latent class membership. Furthermore, being registered with local or state government, being a member of a professional poultry association, and numerous other firm‐specific characteristics listed previously were also not statistically significant covariates for characterizing latent class membership. The lack of significance of these firm‐specific covariates suggests that there was something systematically different between the southwestern states and the other states.

Previous observations in the literature suggest a possible inverse relationship between average precipitation and WTP for aflatoxin reduction of maize. Marechera and Ndwiga (2015) estimated that Kenyan maize farmers in the driest county sampled were more likely to pay for Aflasafe, if it was commercially available than farmers in the wettest county. However, the county with the highest likelihood of adoption in their study was not the driest county. Oyo and Kwara States are wetter than Bauchi, Benue, Kaduna, and Nasarawa States. Hence, the result that enterprises in Bauchi, Benue, Kaduna, and Nasarawa had a higher probability of having higher mean WTP for verified aflatoxin‐reduced maize than farmers in Oyo and Kwara States reinforces the loose trend of higher WTP in regions with lower rainfall.

Geographic differences could stem from differences in culture or agricultural markets. Most of the sampled areas of Oyo and Kwara States are, being predominately Yoruba, distinct ethnically and linguistically from the other states (Uchendu, 2010). It may be that poultry farmers simply are not as concerned about aflatoxin contamination in Oyo and Kwara States as in other states. The southwestern states have a good reputation as poultry farmers and most of Nigeria’s large feed manufacturers are located there. Poultry farmers in Oyo and Kwara States may perceive that many agribusinesses control for aflatoxin (Table 2), believe that those control mechanisms are effective, and—therefore—do not have as strong of a price response to aflatoxin contamination levels. It also could be that poultry farmers in Oyo and Kwara States may have more alternative grain sources to domestically‐produced maize for feeding animals. However, the data in this study was not structured to provide insight into the merits of any of these explanations. Identifying the precise factors driving differences in agribusiness enterprise probability of latent class membership among these states is a promising area for future study. It is very interesting to compare this result to the result of the maize farmer survey that maize farmers in Oyo State were less likely to persist in using Aflasafe than maize farmers in Kaduna State (Johnson et al., 2018).

It is interesting that enterprises aware of aflatoxin had a higher probability of being in Latent Class 2, which has lower mean WTP’s for verified aflatoxin‐safe maize, than enterprises who were not aware. There are three possible explanations of this effect. (a) Enterprise managers who are aware of the damage aflatoxin causes respond not by offering a price premium for aflatoxin‐safe maize but by discounting regular, untested market maize. Such an explanation would be paralleled by De Groote et al.’s findings for Kenyan consumers presented in Section 1. (b) Respondents who were aware of aflatoxin provided less biased survey results. (c) Respondents who were aware of aflatoxin assumed that their current strategies for controlling it were adequate.

It may be that enterprises with aflatoxin awareness had more accurate understandings of the economic benefits of aflatoxin‐safe maize than enterprises with no aflatoxin awareness. Su, Adam, Lusk, and Arthur (2011) suggested that choice experiment respondents with more experience using a product had more stable WTP estimates when different elicitation methods (i.e., choice experiments and experiment auctions) were used. Along a similar line of logic, it may also be that novel threats elicit stronger responses than new threats. This all might suggest that respondents with experience produce less biased results, which would further suggest that the Class 2 estimates may be a better reference for forming long‐run WTP estimations than Class 1.

Alternatively, managers that had heard of aflatoxin may have assumed that their management strategies sufficiently mitigated the problems of aflatoxin contamination. As illustrated in the third data column of Table 2, nearly 75% of enterprises whose survey respondents had heard of aflatoxin also took steps to control for aflatoxin, typically by using toxin binder. When mixed with animal feed, clay minerals can bind to aflatoxin and reduce or prevent aflatoxin from being absorbed by animals’ bodies (Phillips, Afriyie‐Gyawu, Wang, Williams., & Huebner, 2006). However, Hell et al. (2008, p. 226) note that these clay binders “act more as prophylactics than as curative remedies.” If this hypothesis is correct, merely informing more Nigerian agribusiness managers about aflatoxin would only be a starting point for aflatoxin education efforts. Such efforts would specifically need to highlight the virtues of purchasing verified aflatoxin‐safe maize relative to using toxin binder.

## 6 CONCLUSIONS AND IMPLICATIONS

In this analysis it was discovered that less than half of agribusiness enterprise representatives (42.4%) had heard of aflatoxin and only 13% of enterprise representatives had heard of Aflasafe. Geography was determined to be a major factor related to aflatoxin awareness and mitigation. Differences across states in regard to sophistication in controlling for aflatoxin seemed to parallel differences across states in having heard of aflatoxin. For example, in the southwestern states of Oyo and Kwara, 92.2% and 46.0% of enterprises, respectively, controlled for aflatoxin in maize supplies at the time of the survey, the highest rates of any states. These are the same two states with the highest percentages of respondents who had heard of aflatoxin. Geographic differences could stem from differences in culture or agricultural markets. Thus, implications of Aflasafe availability for agribusinesses and end consumers (and thus impacts on human health) are also likely to vary throughout the supply chain and across geographies.

A cultural change will be needed to get to the point where agribusiness enterprises are controlling for aflatoxin (which is needed for improved health). Testing will be one of the first steps. While testing may seem a rather obvious solution to contaminated maize, implications of testing are expected to vary depending on the cost, reliability, and accessibility of such testing. Where toxin binders are commonly used, for example, the results of implementing testing may be harder to measure and discern, at least in the short run because consumers are already being buffered from the negative effects of aflatoxin. Culturally, within the agribusiness community, those areas in which agribusinesses are employing toxin binders may see little reason to adopt widespread testing. Thus, the implications for businesses are largely dependent on the starting point for aflatoxin detection and/or related risk mitigation in that agribusiness itself, within the associated supply chain, and culturally in the region.

If testing develops without Aflasafe usage, feed millers will face the dilemma of what to do with maize with high aflatoxin concentrations. They will either have to discard the maize at high private and social cost or treat the maize with toxin binder. Because toxin binder does not completely mitigate the effect of aflatoxin, the latter approach would result in continued human and livestock aflatoxin exposure. Aflatoxin education efforts targeted at agribusiness managers could emphasize the merits of purchasing verified aflatoxin‐reduced maize over managers’ current practices for controlling aflatoxin contamination and over using toxin binder. At the same time, widespread Aflasafe usage likely cannot develop without testing, because grain buyers can only offer a premium for maize growth with Aflasafe if they have a way of verifying the aflatoxin and Aflasafe content of the maize. Aflatoxin testing and Aflasafe usage by maize farmers need to develop in tandem to generate the greatest benefit for Nigerian poultry farmers, feed millers, and consumers.

## Footnotes

1 When compared with the conventional method of testing statistical significance, the method of comparing overlapping confidence intervals is a more conservative test of significance when the null hypothesis is true and incorrectly fails to reject the null hypothesis more frequently when the null hypothesis is false (Schenker & Gentleman, 2001).
