# Supplementary material for: Willingness to pay of Nigerian poultry producers and feed millers for aflatoxin‐safe maize
Source: Agribusiness (N Y N Y). 2019 Jul 9:21621. doi: 10.1002/agr.21621 (PMC6990872; doi:10.1002/agr.21621)
Supplement: Supplementary file 1 [file AGR-2019-21621-S1.docx]

**Willingness to Pay of Nigerian Poultry Farmers and Feed Millers for Aflatoxin-Safe Maize**

**Online Appendix**

**Questionnaire on Choice Aflasafe for Poultry Owners**

**A. IDENTIFICATION**

| **VARIABLE** | | | | |
| --- | --- | --- | --- | --- |
| State: | | | | |
| LGA: | | | | |
| Town: | | | | |
| Enumerator’s Name: | | | | |
| Name of the enterprise: | | | | |
| Name of the respondent: | | | | |
| Title of the respondent: | | | | |
| Telephone number of the enterprise: | | | | |
| Questionnaire ID: | | | | |
| Date: DD_____MM_____YY | | | | |
| Start time: | | | | |
| GPS coordinates | Waypoint : | N(S) | E(W) | Altitude |

**Script Read to Respondent:**

“Maize that is contaminated with aflatoxin can result in disease and death in poultry. Maize that is treated with Aflasafe results in aflatoxin-safe maize. Different national governments have set different safety levels. The USA level is 20 parts per billion (ppb) or lower. The EU level is 4 ppb or lower. The Nigerian level is 10 ppb or lower. Kindly see below to see how much you will be willing to pay and level of aflatoxin you can tolerate.”

**GENERAL CHARACTERISTICS OF THE ENTERPRISE**

## Name of head of the enterprise: ______________________________

## Name of respondent______________________________________

## What is your position in the enterprise? _________________________

## Does your enterprise make use of maize grain? 1= Yes 0= No

## Age of the head:______________ years

## Gender of the head: 1= Male, 0= Female

## Marital status of the head 1= Single 2= Married 3= Others

## Level of education of the head: 1= Formal Education 0= No formal education

## Number of years of education of respondent______________ years

## What is the legal status of the enterprise? 1=Registered; 2=Unregistered; 3=Other (Specify)__________________

## What is the type of the enterprise? 1= Sole proprietor; 2= Partnership; 3= Cooperative/ Association; 4 = Private Limited Co;5= Public company;6=Joint (private & public);7= Other (Specify)__________________

## Would you characterize your enterprise as small, medium, or large based on the quantity of maize you use per annum. 1= Small scale (<10tons) 2=Medium scale (10 -100tons) 3=Large scale >100tons)

## What type of product do you make? 1. Poultry & feeds 2. Feeds alone 3. Poultry alone 4. Maize based food products

## For how many years has your enterprise been operating? _______________________

## Is your operation registered with NAFDAC (0=No 1=Yes)

## Is your operation registered with Local/State government? (0=No 1=Yes)

## Net revenue per year for the enterprise N______________

## Does your enterprise belong to any poultry associations or professional body? (1 = Yes, 0 = No)

## Does your enterprise belong to any feed miller associations or professional body? (1 = Yes, 0 = No)

## If yes for how many years have it been a member? _____

## Does your enterprise have access to micro-credit for boosting business? (Yes.=1; No = 0)

**Agribusiness Block 1**

**1. Please select the option you would choose from the following scenario**

| **Option A** |  | **Option B** |  | **Option C** |
| --- | --- | --- | --- | --- |
| Price premium over regular market price for maize | 41-50% | Price premium over regular market price for maize | 41-50% |  |
| Level of Aflatoxin in Maize | 20PPB | Level of Aflatoxin in Maize | 4PPB | I choose to purchase regular maize from market (not tested for aflatoxin) |

**2. Please select the option you would choose from the following scenario**

| **Option A** |  | **Option B** |  | **Option C** |
| --- | --- | --- | --- | --- |
| Price premium over regular market price for maize | 31-40% | Price premium over regular market price for maize | 1-10% |  |
| Level of Aflatoxin in Maize | 10PPB | Level of Aflatoxin in Maize | 4PPB | I choose to purchase regular maize from market (not tested for aflatoxin) |

**3. Please select the option you would choose from the following scenario**

| **Option A** |  | **Option B** |  | **Option C** |
| --- | --- | --- | --- | --- |
| Price premium over regular market price for maize | 21-30% | Price premium over regular market price for maize | 1-10% |  |
| Level of Aflatoxin in Maize | 20PPB | Level of Aflatoxin in Maize | 10PPB | I choose to purchase regular maize from market (not tested for aflatoxin) |

**4. Please select the option you would choose from the following scenario**

| **Option A** |  | **Option B** |  | **Option C** |
| --- | --- | --- | --- | --- |
| Price premium over regular market price for maize | 0% | Price premium over regular market price for maize | 21-30% |  |
| Level of Aflatoxin in Maize | 10PPB | Level of Aflatoxin in Maize | 10PPB | I choose to purchase regular maize from market (not tested for aflatoxin) |

**5. Please select the option you would choose from the following scenario**

| **Option A** |  | **Option B** |  | **Option C** |
| --- | --- | --- | --- | --- |
| Price premium over regular market price for maize | 1-10% | Price premium over regular market price for maize | 41-50% |  |
| Level of Aflatoxin in Maize | 10PPB | Level of Aflatoxin in Maize | 20PPB | I choose to purchase regular maize from market (not tested for aflatoxin) |

**6. Please select the option you would choose from the following scenario**

| **Option A** |  | **Option B** |  | **Option C** |
| --- | --- | --- | --- | --- |
| Price premium over regular market price for maize | 11-20% | Price premium over regular market price for maize | 41-50% |  |
| Level of Aflatoxin in Maize | 4PPB | Level of Aflatoxin in Maize | 10PPB | I choose to purchase regular maize from market (not tested for aflatoxin) |

**7. Please select the option you would choose from the following scenario**

| **Option A** |  | **Option B** |  | **Option C** |
| --- | --- | --- | --- | --- |
| Price premium over regular market price for maize | 11-20% | Price premium over regular market price for maize | 11-20% |  |
| Level of Aflatoxin in Maize | 10PPB | Level of Aflatoxin in Maize | 4PPB | I choose to purchase regular maize from market (not tested for aflatoxin) |

**Agribusiness Block 2**

**1. Please select the option you would choose from the following scenario**

| **Option A** |  | **Option B** |  | **Option C** |
| --- | --- | --- | --- | --- |
| Price premium over regular market price for maize | 41-50% | Price premium over regular market price for maize | 31-40% |  |
| Level of Aflatoxin in Maize | 10PPB | Level of Aflatoxin in Maize | 10PPB | I choose to purchase regular maize from market (not tested for aflatoxin) |

**2. Please select the option you would choose from the following scenario**

| **Option A** |  | **Option B** |  | **Option C** |
| --- | --- | --- | --- | --- |
| Price premium over regular market price for maize | 31-40% | Price premium over regular market price for maize | 11-20% |  |
| Level of Aflatoxin in Maize | 4PPB | Level of Aflatoxin in Maize | 10PPB | I choose to purchase regular maize from market (not tested for aflatoxin) |

**3. Please select the option you would choose from the following scenario**

| **Option A** |  | **Option B** |  | **Option C** |
| --- | --- | --- | --- | --- |
| Price premium over regular market price for maize | 21-30% | Price premium over regular market price for maize | 0% |  |
| Level of Aflatoxin in Maize | 10PPB | Level of Aflatoxin in Maize | 20PPB | I choose to purchase regular maize from market (not tested for aflatoxin) |

**4. Please select the option you would choose from the following scenario**

| **Option A** |  | **Option B** |  | **Option C** |
| --- | --- | --- | --- | --- |
| Price premium over regular market price for maize | 0% | Price premium over regular market price for maize | 31-40% |  |
| Level of Aflatoxin in Maize | 20PPB | Level of Aflatoxin in Maize | 4PPB | I choose to purchase regular maize from market (not tested for aflatoxin) |

**5. Please select the option you would choose from the following scenario**

| **Option A** |  | **Option B** |  | **Option C** |
| --- | --- | --- | --- | --- |
| Price premium over regular market price for maize | 0% | Price premium over regular market price for maize | 1-10% |  |
| Level of Aflatoxin in Maize | 4PPB | Level of Aflatoxin in Maize | 20PPB | I choose to purchase regular maize from market (not tested for aflatoxin) |

**6. Please select the option you would choose from the following scenario**

| **Option A** |  | **Option B** |  | **Option C** |
| --- | --- | --- | --- | --- |
| Price premium over regular market price for maize | 1-10% | Price premium over regular market price for maize | 0% |  |
| Level of Aflatoxin in Maize | 20PPB | Level of Aflatoxin in Maize | 10PPB | I choose to purchase regular maize from market (not tested for aflatoxin) |

**7. Please select the option you would choose from the following scenario**

| **Option A** |  | **Option B** |  | **Option C** |
| --- | --- | --- | --- | --- |
| Price premium over regular market price for maize | 11-20% | Price premium over regular market price for maize | 21-30% |  |
| Level of Aflatoxin in Maize | 20PPB | Level of Aflatoxin in Maize | 20PPB | I choose to purchase regular maize from market (not tested for aflatoxin) |

# INFORMATION AWARENESS & COMMUNICATION

## Have you heard about aflatoxin? (0=No 1=Yes)

**If yes, answer questions in the table below**

| **Variable** | **1=Yes, 2=No, 3= Don’t know** |
| --- | --- |
| Can feeding aflatoxin contaminated maize increase mortality in your chicks? |  |
| Can feeds made of aflatoxin contaminated maize reduce the quantity of eggs produced by your chicken? |  |
| Can eating food product made from aflatoxin contaminated maize be bad for consumers’ health? |  |
| Can eating food product made from aflatoxin contaminated maize contribute to ‘stunted growth’ in children? |  |

## Have you been testing for aflatoxin level in your maize supply? (0=No 1=Yes)

## If you are testing for aflatoxin, what testing methods do you use? ______________________________________

## Do you control for aflatoxin on your maize products? (0=No 1=Yes)

## If you control for aflatoxin in your maize products, please describe what you do. ____________________________________________________________________________________________________________________________________________________________

## Did you have experience paying a price premium for delivery of aflatoxin reduced maize? (0=No 1=Yes)

## If yes, indicate the price premium in below table

**Price Premium**

| **Years** | **What price premium did you pay** | **`Maize quality** |
| --- | --- | --- |
| 2015 – Lean season | _______% | - 4 ppb - 10 ppb - 20 ppb - Not tested |
| 2015 – Planting season | _______% | - 4 ppb - 10 ppb - 20 ppb - Not tested |

## Have you heard about Aflasafe? (0=No 1=Yes)

If yes, attend to the table below

| **Variable** | **Response** |
| --- | --- |
| When did you first hear about it?(e.g. 2001) |  |
| Where did you hear about it?(Code B: TV, Radio, another farmers, billboard, IITA, ADP, Company, others)check all that apply |  |

## **From the table below, what months do you typically buy maize?**

| **Months** | **1=Yes, 0=No** |
| --- | --- |
| November |  |
| December |  |
| January |  |
| February |  |
| March |  |
| April |  |
| June |  |
| July |  |
| August |  |
| September |  |
| October |  |
| November |  |
| December |  |

## **Relationship with implementer (see the table below)**

| **Years** | **Are you an implementer with AgResults?Yes=1, no=0** | **Are you working with an implementer (yes or no)** | **If yes, Name of implementer** |
| --- | --- | --- | --- |
| 2016 |  |  |  |
| 2015 |  |  |  |
| 2014 |  |  |  |
| 2013 |  |  |  |
| 2012 |  |  |  |
